# Differential assembly of root-associated bacterial and fungal communities of a dual transgenic insect-resistant maize line at different host niches and different growth stages

**DOI:** 10.3389/fmicb.2022.1023971

**Published:** 2022-09-29

**Authors:** Zhongling Wen, Weixuan Yao, Mi Han, Xinhong Xu, Fengci Wu, Minkai Yang, Aliya Fazal, Tongming Yin, Jinliang Qi, Guihua Lu, Rongwu Yang, Xinyuan Song, Yonghua Yang

**Affiliations:** ^1^State Key Laboratory of Pharmaceutical Biotechnology, Institute for Plant Molecular Biology, School of Life Sciences, Nanjing University, Nanjing, China; ^2^Co-innovation Center for Sustainable Forestry in Southern China, Nanjing Forestry University, Nanjing, China; ^3^Jilin Provincial Key Laboratory of Agricultural Biotechnology, Agro-Biotechnology Research Institute, Jilin Academy of Agricultural Sciences, Changchun, China; ^4^School of Life Sciences, Huaiyin Normal University, Huaian, China

**Keywords:** differential assembly, microbial communities, genetically modified maize, host niches, growing stages

## Abstract

Transgenic technology has been widely applied to crop development, with genetically modified (GM) maize being the world’s second-largest GM crop. Despite the fact that rhizosphere bacterial and fungal populations are critical regulators of plant performance, few studies have evaluated the influence of GM maize on these communities. Plant materials used in this study included the control maize line B73 and the *mcry1Ab* and *mcry2Ab* dual transgenic insect-resistant maize line 2A-7. The plants and soils samples were sampled at three growth stages (jointing, flowering, and maturing stages), and the sampling compartments from the outside to the inside of the root are surrounding soil (SS), rhizospheric soil (RS), and intact root (RT), respectively. In this study, the results of alpha diversity revealed that from the outside to the inside of the root, the community richness and diversity declined while community coverage increased. Morever, the different host niches of maize rhizosphere and maize development stages influenced beta diversity according to statistical analysis. The GM maize line 2A-7 had no significant influence on the composition of microbial communities when compared to B73. Compared to RS and SS, the host niche RT tended to deplete Chloroflexi, Gemmatimonadetes and Mortierellomycota at phylum level. Nitrogen-fixation bacteria *Pseudomonas*, *Herbaspirillum huttiense*, *Rhizobium leguminosarum*, and *Sphingomonas azotifigens* were found to be enriched in the niche RT in comparison to RS and SS, whilst *Bacillus* was found to be increased and *Stenotrophomonas* was found to be decreased at the maturing stage as compared to jointing and flowering stages. The nitrogen fixation protein FixH (clusters of orthologous groups, COG5456), was found to be abundant in RT. Furthermore, the pathogen fungus that causes maize stalk rot, *Gaeumannomyces radicicola*, was found to be abundant in RT, while the beneficial fungus *Mortierella hyalina* was found to be depleted in RT. Lastly, the abundance of *G. radicicola* gradually increased during the development of maize. In conclusion, the host niches throughout the soil-plant continuum rather than the Bt insect-resistant gene or Bt protein secretion were primarily responsible for the differential assembly of root-associated microbial communities in GM maize, which provides the theoretical basis for ecological agriculture.

## Introduction

Genetically modified (GM) crops are crops whose genome can be modified through genetic engineering techniques to improve existing traits or introduce a new trait, which usually involves inserting specific fragments of exogenous nucleic acid/gene sequence into their genome to ensure that they can achieve one or more complex characteristics (Kumar et al., 2020). With 60.9 million hectares, GM maize is one of the most popular biotech crops, accounting for 32% of the global biotech crop land (ISAAA, 2019). Genetic modifications can help host plants in gaining a growth advantage under biotic and abiotic stress. For example, grain borer and maize weevil can cause 14–36% grain losses; however, the use of high yielding insect resistant maize varieties could significantly reduce the losses (Tefera et al., 2016).

The interactions between plants and the environment are widespread in nature, and one of the most important forms is the interaction between plants and rhizosphere microorganisms (Paterson et al., 2007; Bulgarelli et al., 2013; Ofek et al., 2014). Plant rhizosphere microbial communities, including bacteria and fungi, participate in the whole life cycle of plants and affect the nutrition, growth, and development and environmental adaptability of host plants, while the host plants can strongly influence the composition, structure, and function of rhizosphere microbiota through the litter of plant or root exudates in return (Muller et al., 2016; Martin et al., 2017; Duran et al., 2018; Kwak et al., 2018; Zhang et al., 2019).

With the increasing benefits of the wide commercial application of GM crops, people are also paying more and more attention to the impact of GM crops on the surrounding environment, especially on the root-associated microbial communities (Saxena and Stotzky, 2001; Tefera et al., 2016; Mandal et al., 2020). The large-scale planting of GM crops can change the rhizosphere microbial communities by releasing transgenic products into the rhizosphere through root exudates or plant degradation, or transferring and integrating plant DNA into microorganisms through horizontal gene transfer (Saxena and Stotzky, 2001; Berendsen et al., 2012; Doornbos et al., 2012; Mandal et al., 2020). Maize is one of the main crops in the world. Bt insect-resistant maize is widely used in the world, and the root-associated microbial communities may possibly be affected by introducing Bt toxin protein into the soil (Prihoda and Coats, 2008). GM Bt crops produce a protein-like crystalline substance known as Bt-toxin, which can reduce crop damage due to insect attacks by causing toxemia and death of insects (Singh and Dubey, 2016; Mandal et al., 2020). Bt toxin protein has been used in agricultural production as a sprayable biological insecticide for decades (Mandal et al., 2020). Pesticide resistance is a major agricultural concern in crop production, with over 500 insects resistant to standard pesticides, but GM Bt crops can minimize insecticide use while protecting non-target insect diversity (Mandal et al., 2020). By introducing the *mcry1Ab* and *mcry2Ab* genes into maize, the host plant can produce Bt-toxin, which provides insect resistance (Liliana et al., 2012; Fazal et al., 2020). The protein in Bt toxins may adsorb and mix with clay, causing severe harm to soil microbes (Strain and Lydy, 2015; Li et al., 2018). Some prior studies found that GM Bt crops had no effect on soil microbial enzymes and properties, whereas other studies found that GM Bt crops had a substantial impact on rhizosphere soil microbial properties and enzymatic activity, as well as the gram-positive to gram-negative bacteria ratio (Xue et al., 2005; Coz et al., 2008; Chen et al., 2012).

Plant developmental stage and host niches also driven the differentiation in ecological role of the maize microbiome. A recent study suggested that maize developmental stage had a strong influence on the microbial diversity, composition and interkingdom networks in plant compartments by enriching some beneficial bacterial bacteria such as Actinobacteria, Burkholderiaceae and Rhizobiaceae at the early stage, and enriching more saprophytic fungi at the late stage (Xiong et al., 2021). Community composition also varies significantly between different host niches. For example, as compared to the rhizosphere, the plant endophytic layer tended to enrich Proteobacteria, Firmicutes and Bacteroidetes, and deplete Acidobacteria, Planctomycetes, Chloroflexi, and Verrucomicrobia (Trivedi et al., 2021). Due to the assembly and shift of a plant-associated microbiome is a successional and complex process that is determined by species interactions, the environment, the developmental stage and host niches (Trivedi et al., 2021), the design containing multiple plant developmental stage and sampling compartment is practicable and effective to comprehensively explore the differential of plant microbial communities.

In this study, the plant materials employed were the control maize line B73 and the GM maize line 2A-7, and the basic physicochemical properties of soils and plants were analyzed by comparing their element contents and soil enzyme activities. High-throughput sequencing of V3-V4 hypervariable regions of the 16S rRNA gene and ITS1 regions of ITS gene amplicons was performed and evaluated using the Illumina MiSeq at three different sampling stages and compartments (i.e., host niches of maize rhizosphere). This work is expected to give a scientific basis for breeding in ecological agriculture by predicting the functions of microbiomes based on their classification.

## Materials and methods

### Plant materials and sampling methods

The control maize line was B73, and the transgenic insect-resistant maize line was 2A-7 (produced by the insertion of the *mcry1Ab* and *mcry2Ab* genes). The experimental field was located in Gongzhuling City, Jilin Province, China (124°82′E, 43°52′N). The climate conditions of the experimental location were a sub-humid warm continental monsoon climate and the soil type belonged to the rust yellow black soil, a phylum of meadow black soil subclass according to the information obtained from the China Soil Database.^1^ This field was divided into 6 plots (10 m × 15 m per plot), and each of the above two maize treatments had three replications. Then the sampling steps were done by using the method previously described with modifications (Inceoglu et al., 2010; Lu et al., 2018). To avoid false environments, it is critical to avoid the root at the interface of the plot and the soil during sampling (Peiffer et al., 2013). After excavation, plants and soil samples were immediately placed in a plastic bag with several pre-freezing chemical ice packs and then taken to the laboratory. At all sampling stages, every plant and soil of one treatment was sampled from three different plots with five sampling points per plot. The five sampling points were randomly distributed across the plot, and composite samples from five sampling points were made and treated as one replicate.

Experimental samples were collected at the plant before planting on May 28, the jointing stage on July 13, the flowering stage on August 11, and the maturing stage on September 28 in 2019. The “bulk soil samples” (BS) was only sampled at the plant before planting. From the outside to the inside of the root, soil loosely adhering to the roots was shaken off from the maize plant as surrounding soil (SS). Rhizospheric soil (RS) was then collected after brushing with phosphate buffered saline (PBS). The roots were then further washed with PBS and 4,000 g centrifugal and collected by grinding with liquid nitrogen (mixed samples of rhizoplane/endosphere, RT) (Yang et al., 2020). SS, RS, and RT were sampled at the jointing, flowering and maturing stages. The Center of Modern Analysis at Nanjing University then measured basic soil and plant data. All the samples were stored at −80°C in a freezer before DNA extraction.

The soil total carbon and nitrogen content were measured by the Center of Modern Analysis at Nanjing University using bulk soils. The enzyme activities of sucrase (S-SC, EC 3.2.1.26), nitrate reductase (S-NR, EC 1.7.99.4), nitrite reductase (S-NiR, EC 1.7.99.3), urease (S-UE, EC 3.5.1.5), acid phosphatase (S-ACP, EC 3.1.3.2), and alkaline phosphatase (S-AKP/ALP, EC 3.1.3.1) were measured by corresponding kits purchased from Solarbio (Beijing Solarbio Science & Technology Co., Ltd) by using RS filtered through a 50-μm mesh after natural air drying.

### DNA extraction from soil and root samples

In this study, approximately 0.30 g of all soil and root samples of every biological replicate were used to extract total metagenomic DNA by using the MoBio PowerSoil DNA Isolation Kit (MoBio Laboratories Inc., Carlsbad, CA, USA), following the instructions with minor modifications by homogenizing samples in lysis buffer by using a tissue grinder (Grinder-48, Gallop technology) at 60 Hz for 600 s (Lu et al., 2017; Wen et al., 2019, 2020). After extraction, the quality of DNA samples was assessed on a 1% agarose gel, and then subsequently quantified by using a Qubit Fluorometer (Qubit 2.0, Invitrogen, Carlsbad, CA, USA) to ensure they were more than 10 ng/μl (Kennedy et al., 2014).

### DNA amplicon sequencing and clean data

The DNA extraction samples were sent to Majorbio Bio-pharm Technology Co., Ltd (Shanghai, China) for subsequent PCR amplification, product purification, library quality determination, and high-throughput sequencing. The approximately 468 bp encompassing the V3–V4 hypervariable region of the 16S rRNA and approximately 350 bp encompassing the internal transcribed spacer (ITS1 region) were amplified by primer pair 338F/806R (Xu et al., 2016) and ITS1F/ITS2R (Adams et al., 2013), respectively. The Sequence Read Archive (SRA) accession numbers for 16S rRNA sequencing and ITS sequencing clean data are PRJNA785383 and PRJNA785713, respectively.

### Amplicon sequencing data and statistical analyses

The analysis of 16S rRNA amplicon sequencing data and ITS amplicon sequencing data was based on the databases silva 138/16s^2^ and Unite 8.0/ITS_fungi,^3^ respectively. All data were sub-sampled according to the minimum sample number sequence in Majorbio^4^ using the software R (v3.1.3), and the analysis of 16S rRNA amplicon sequencing data also excluded data from the phylum Cyanobacteria and family Mitochondria to remove the mitochondrial and chloroplast sequences.

Alpha diversity was analyzed through the observed OTUs index, Shannon index, and Coverage index to reflect the community’s richness, diversity, and coverage, while Beta diversity was conducted through principal component analysis (PCA), principal co-ordinates analysis (PCoA), non-metric multidimensional scaling analysis (NMDS), and partial least squares discriminant analysis (PLS-DA) to evaluate differences in species complexity (Schloss et al., 2009; Caporaso et al., 2010). Then the analysis of similarities (ANOSIM) and PERMANOVA analysis (Adonis) were performed by using the software R (v3.1.3) (Zhou et al., 2011). The analysis of functional genes was performed by using the software PICRUSt and the Fungi Functional Guild (FUNGuild). All these analyses of sequencing data above were performed on the platform Majorbio (see text footnote 4).

### Statistical analyses

One-way ANOVA was used to evaluate the selected properties of soils and plants, alpha diversity indices, the quantification of *nifH* gene, and the abundance of species and functional genes by using software GraphPad Prism 8. The analysis of similarities (ANOSIM) and PERMANOVA analysis (Adonis) were performed by using the software R (v3.1.3) in platform Majorbio based on the Bray-Curtis distance metric.

## Results

### Basic physicochemical properties of soils and plants

**FIGURE 1:**
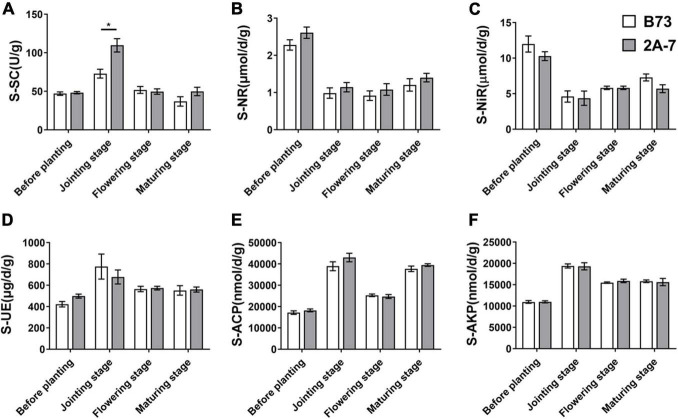
The activities of six key enzymes involved in the carbon, nitrogen and phosphorus cycles of root-associated microbial communities. B73 and 2A-7 represent the control maize line B73 and the transgenic insect tolerant maize line 2A-7, respectively. S-SC, S-NR, S-NiR, S-UE, S-ACP and S-AKP represent sucrase **(A)**, nitrate reductase **(B)**, nitrite reductase **(C)**, urease **(D)**, acid phosphatase **(E)** and alkaline phosphatase **(F)**, respectively.

There were no significant differences in soil pH, water content, carbon (C) content, nitrogen (N) content, or C:N ratios between the B73 and 2A-7 groups (Table 1). Neither the C nor N content of plants differed significantly between these two groups (Table 1). Additionally, the water content of soil varied across four different sampling stages (*p* < 0.05). Moreover, the maize C content was significantly higher and the maize N content was significantly lower during the maturing stage compared to the jointing and flowering stages (Table 1). When the enzyme activities closely related to C, N, and phosphorus (P) cycles were compared, there existed no significant difference between the B73 and 2A-7 groups in the activities of nitrate reductase, nitrite reductase, urease, acid phosphatase and alkaline phosphatase (Figure 1). However, when compared to B73, the enzyme activities of sucrase were significantly higher in 2A-7 groups at the jointing stage (Figure 1A).

**TABLE 1:** Physicochemical properties of soil and plant.

	Sampling stages	Before planting (Mean ± SD)		Jointing stage (Mean ± SD)		Flowering stage (Mean ± SD)		Maturing stage (Mean ± SD)	
	
	Samples	B73	2A-7	B73	2A-7	B73	2A-7	B73	2A-7
**Soil analysis**	pH value	7.30 ± 0.13	7.36 ± 0.16	7.17 ± 0.22	7.14 ± 0.10	6.51 ± 0.50	6.58 ± 0.27	6.65 ± 0.29	6.56 ± 0.10
	Water content (%)	21.03 ± 1.02	21.90 ± 0.38	16.10 ± 1.30	17.58 ± 2.89	23.22 ± 1.35	23.68 ± 1.31	18.96 ± 0.77	18.42 ± 1.87
	C content (%)	1.52 ± 0.17	1.55 ± 0.11	1.53 ± 0.15	1.54 ± 0.10	1.64 ± 0.13	1.60 ± 0.11	1.52 ± 0.14	1.52 ± 0.11
	N content (%)	0.14 ± 0.01	0.13 ± 0.01	0.15 ± 0.01	0.14 ± 0.00	0.15 ± 0.18	0.15 ± 0.01	0.14 ± 0.00	0.15 ± 0.01
	C:N ratios	11.10 ± 0.35	12.25 ± 1.46	10.43 ± 0.38	11 ± 0.68	10.99 ± 0.42	10.65 ± 0.21	10.83 ± 1.02	10.38 ± 0.34
**Plant analysis**	C content (%)	NA	NA	37.10 ± 2.06	39.18 ± 0.78	38.80 ± 0.24	39.14 ± 0.24	**42.02 ± 0.14**	**42.10 ± 0.08**
	N content (%)	NA	NA	1.64 ± 0.07	1.72 ± 0.19	1.86 ± 0.05	1.79 ± 0.04	**1.24 ± 0.03**	**1.28 ± 0.05**

Values were mean ± SD (n = 3). SD represents standard deviation. B73 and 2A-7 represent the control maize line B73 and the transgenic insect tolerant maize line 2A-7, respectively. C content and N content represent carbon content and nitrogen content. Soil analysis using the bulk soil before planting and surrounding soil at the jointing, flowering and maturing stages.

The significance test was performed by using one-way ANOVA.

The values in bold indicate the significant difference (p < 0.05) between B73 and 2A-7 groups by the tests.

### Alpha and beta diversity of root-associated microbial communities

**FIGURE 2:**
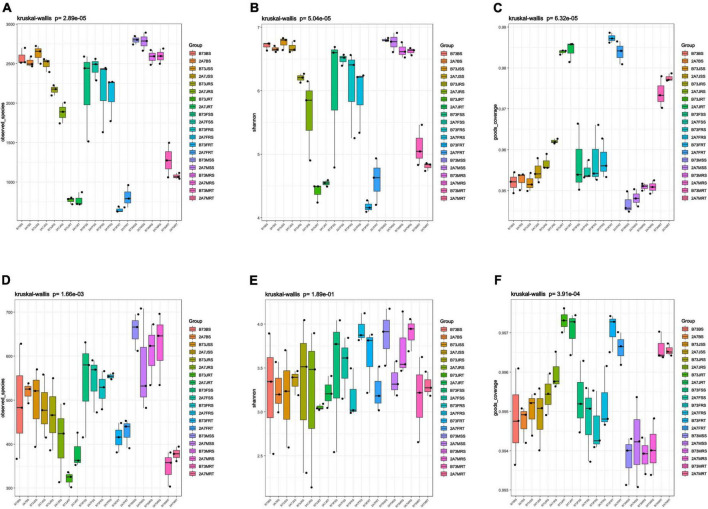
The boxplot of alpha diversity of bacterial community **(A–C)** and fungal community **(D–F)**. The results of alpha diversity through three different indices (Observed species value, Shannon value, and Good’s coverage) were divided in two groups according to different sequencing results. B73 and 2A7 represent the control maize line B73 and the transgenic insect tolerant maize line 2A-7, respectively. J, F, and M represent the jointing, flowering, and maturing stages of maize, respectively. BS, SS, RS, and RT represent the bulk soil before planting, surrounding soil, rhizospheric soil and root samples, respectively. The mean and standard Error of Mean (SE) were calculated based on three alpha diversity indices of all replicates and the one-way ANOVA was used for multigroup comparisons.

Supplementary Table 1 contains information on 16S rRNA clean reads and ITS amplicon sequencing data. In the bacterial (Figures 2A,C,E) and fungal communities (Figures 2B,D,F), the values of Observed OTUs, Shannon and Coverage indices showed that there were no significant differences in Alpha diversity between the two varieties or between different sampling stages. However, when compared to SS and RS in both bacterial and fungal communities, the Observed OTUs and Shannon indices were lower and the Coverage index was higher in the niche RT (Figure 2).

According to the Pan/Core OTU analysis, the total species number was 6898/2957, and the core species number was 101/27 in the bacterial/fungal community (Supplementary Table 2). In the PCA, PCoA, NMDS, and PLS-DA charts, there was no significant difference in distance between the control maize line B73 and the transgenic maize line 2A-7 in both bacterial and fungal communities (Supplementary Figure 1). BS, SS, and RS, on the other hand, were clustered together, and they all had a difference in distance between RT (Supplementary Figure 1). Then ANOSIM and Adonis statistical analyses were then performed and the results showed that there existed no difference between the B73 and 2A-7 groups in both bacterial and fungal communities (Table 2). Moreover, the results showed that there was a significant difference between different maize rhizosphere host niches, as well as different growth stages (Table 2).

**TABLE 2:** Significance tests of microbial communities’ structure between samples.

	Group vs. Group	Adonis		ANOSIM	
		*R* ^2^	*P*-value	Statistic	*P*-value
**Bacterial community**	B73BS vs. 2A7BS	0.2003	0.5	−0.037	0.594
	B73JSS vs. 2A7JSS	0.2495	0.1	0.2222	0.414
	B73JRS vs. 2A7JRS	0.2219	0.2	0.0741	0.319
	B73JRT vs. 2A7JRT	0.1449	0.9	−0.2593	1
	B73FSS vs. 2A7FSS	0.1467	1	−0.111	0.908
	B73FRS vs. 2A7FRS	0.1384	0.8	−0.1852	1
	B73FRT vs. 2A7FRT	0.2735	0.2	0.1481	0.39
	B73MSS vs. 2A7MSS	0.1469	0.9	−0.4444	1
	B73MRS vs. 2A7MRS	0.1544	0.8	−0.3704	0.899
	B73MRT vs. 2A7MRT	0.1981	0.5	0.037	0.506
	B73 vs. 2A7	0.006	0.974	−0.0239	0.976
	Jointing vs Flowering	0.0873	**0.016**	0.1432	**0.014**
	Jointing vs. Maturing	0.0893	**0.015**	0.1546	**0.011**
	Flowering vs. Maturing	0.1096	**0.005**	0.1851	**0.007**
	BS vs. SS	0.1112	**0.013**	0.0561	0.295
	BS vs. RS	0.292	**0.001**	0.6055	**0.001**
	BS vs. RT	0.5394	**0.001**	1	**0.001**
	SS vs. RS	0.203	**0.001**	0.5225	**0.001**
	SS vs. RT	0.5463	**0.001**	0.9894	**0.001**
	RS vs. RT	0.368	**0.001**	0.9179	**0.001**
**Fungal community**	B73BS vs. 2A7BS	0.2934	0.2	0.1481	0.39
	B73JSS vs. 2A7JSS	0.2357	0.3	0.1852	0.298
	B73JRS vs. 2A7JRS	0.1861	0.7	−0.037	0.802
	B73JRT vs. 2A7JRT	0.2503	0.3	0.1852	0.302
	B73FSS vs. 2A7FSS	0.0993	1	−0.3333	1
	B73FRS vs. 2A7FRS	0.1564	0.7	−1481	0.903
	B73FRT vs. 2A7FRT	0.4974	0.1	0.8889	0.098
	B73MSS vs. 2A7MSS	0.1827	0.7	−0.037	0.689
	B73MRS vs. 2A7MRS	0.1257	0.9	−0.2963	0.889
	B73MRT vs. 2A7MRT	0.1687	0.8	0	0.478
	B73 vs. 2A7	0.0203	0.273	0.009	0.257
	Jointing vs. Flowering	0.0474	0.089	0.0529	0.085
	Jointing vs. Maturing	0.1231	**0.001**	0.2583	**0.001**
	Flowering vs. Maturing	0.0919	**0.003**	0.1713	**0.006**
	BS vs. SS	0.0931	**0.015**	0.2254	0.05
	BS vs. RS	0.1669	**0.001**	0.4556	**0.001**
	BS vs. RT	0.3047	**0.001**	0.9443	**0.001**
	SS vs. RS	0.1311	**0.001**	0.2988	**0.001**
	SS vs. RT	0.3707	**0.001**	0.9438	**0.001**
	RS vs. RT	0.2195	**0.001**	0.6366	**0.001**

ANOSIM means analysis of similarities and Adonis is used for PERMANOVA (permutational multivariate analysis of variance) based on the Bray–Curtis distance metrics. The *P*-values in bold indicate the significant difference (*p* < 0.05) between groups by the tests. Treatment’s details were as in Figure 1.

### Composition and function of root-associated microbial communities

**FIGURE 3:**
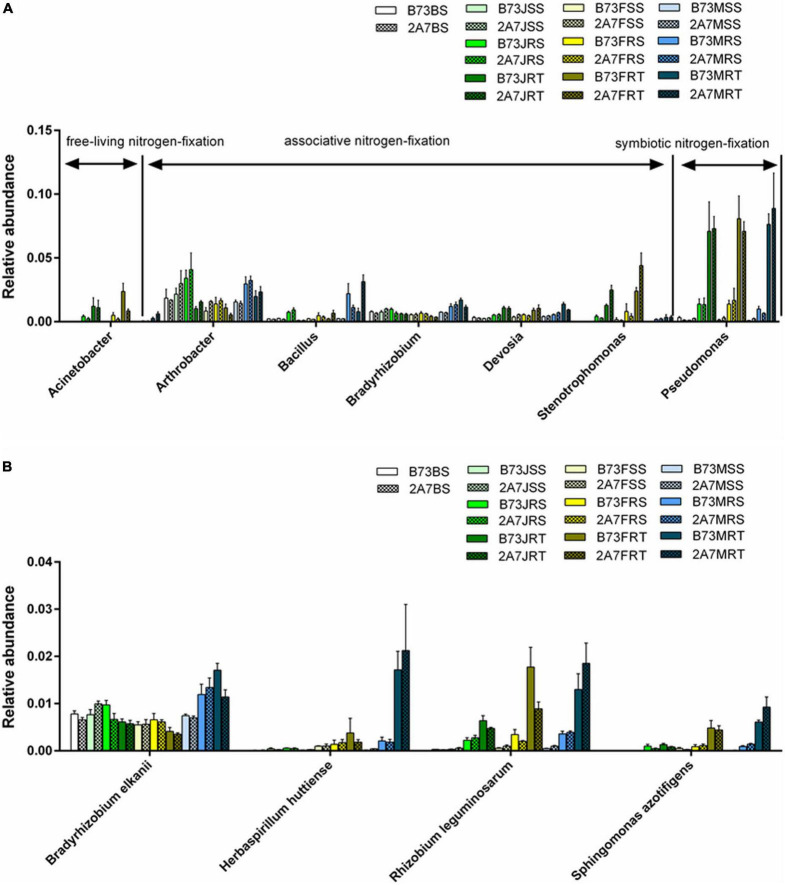
The composition and abundance of bacteria related to nitrogen fixation in maize root-associated bacterial community. **(A)** The abundance of seven nitrogen-fixation bacteria at genus level. **(B)** The abundance of four nitrogen-fixation bacteria at species level. Values are mean ± SD (*n* = 3), treatment’s details were as in Figure 2.

In the bacterial community, Proteobacteria was the most abundant phylum, followed by Actinobacteria, Acidobacteria, Bacteroidetes, and Chloroflexi; while in the fungal community, Ascomycota was the most abundant phylum, followed by Basidiomycota and Mortierellomycota (Supplementary Figure 2). When comparing different host niches, the relative abundance of phyla Chloroflexi, Gemmatimonadetes, and Mortierellomycota in the niche RT was lower than that in the niches RS, SS, and BS, despite no significant differences between B73 and 2A-7 or between different sampling stages (Supplementary Figure 2). At the genus level, seven major nitrogen-fixing bacterial genera were identified, including *Acinetobacter*, *Arthrobacter*, *Bacillus*, *Bradyrhizobium*, *Devosia*, *Stenotrophomonas*, and *Pseudomonas*, whose relative abundance did not differ significantly between B73 and 2A-7 (Figure 3A). However, the relative abundance of *Pseudomonas* was found to be significantly higher in niche RT than in other niches during the jointing, flowering, and maturing stages (Figure 3A). Moreover, the relative abundance of *Bacillus* was higher in maturing stages than in jointing and flowering stages, whereas the relative abundance of *Stenotrophomonas* was lower in maturing stages than in jointing and flowering stages (Figure 3A). In addition, there was no significant difference between the two varieties in the relative abundance of the AMF genus *Funneliformis*, which belongs to the phylum Glomeromycota (Nadimi et al., 2016).

**FIGURE 4:**
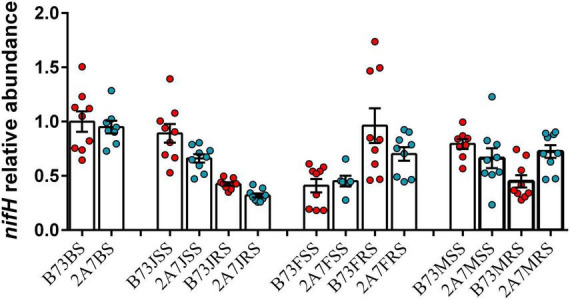
Relative abundance of *nifH* gene in maize root-associated bacterial community of B73 and 2A-7 at different sampling compartments and stages. Value of 1 was assigned to the detected value of the bulk soil of B73 before planting (B73BS). The error bars represent the standard deviation of three replicates of samples and each replicate with technical triplicate, values are mean ± SD (*n* = 9). Treatment’s details were as in Figure 2.

At the species level, *Bradyrhizobium elkanii*, *Herbaspirillum huttiense*, *Rhizobium leguminosarum*, and *Sphingomonas azotifigens* were found to be closely associated to nitrogen-fixation (Figure 3B and Supplementary Table 3). The statistical analysis revealed no significant difference in the relative abundance of four nitrogen-fixing species between two varieties or stages. However, the relative abundance of *H. huttiense*, *R. leguminosarum*, and *S. azotifigens* in the niche RT was significantly higher than in the niches BS, SS and RS, particularly in maturing stage. Further verification was carried out by qPCR, where the copy number of *nifH* gene did not differ significantly between different varieties, sampling stages, or host niches (Figure 4).

All identified fungal species that were either plant pathogens (e.g., *Gaeumannomyces radicicola*, *Fusarium solani*, *Nectria ramulariae*, etc.) or beneficial to plant growth (e.g., *Mortierella hyalina*, *Clonostachys rosea*, and *Chrysosporium pseudomerdarium*) had no significant difference in the relative abundance between B73 and 2A-7 (Supplementary Table 4). Interestingly, when comparing different host niches, the relative abundance of the pathogen *G. radicicola*, which causes maize stalk rot, was lower in the SS than in the RS and RT, while the beneficial root-colonizing fungus *M. hyaline* was enriched in the SS and RS than in the RT. Furthermore, when comparing different stages, only *G. radicicola* was found to gradually enrich with maize growth (i.e., the abundance was highest at the maturing stage) (Supplementary Table 4).

**FIGURE 5:**
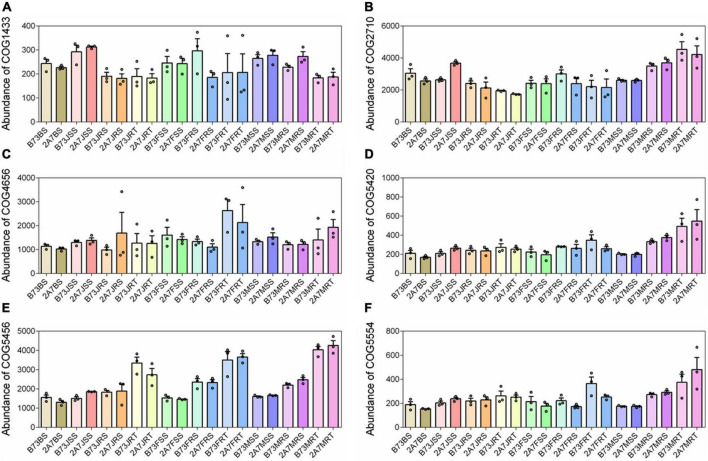
The relative abundance of COG function classification which were directly related to nitrogen-fixation. The six COGs from **(A–F)** were described as dinitrogenase iron-molybdenum cofactor biosynthesis protein **(A)**, nitrogenase **(B)**, required for nitrogen fixation **(C)**, nitrogen fixation **(D)**, nitrogen fixation protein FixH **(E)**, and nitrogen fixation protein **(F)**, respectively. Treatment’s details were as in Figure 2.

Using the software PICRUSt2 and FUNGuild to compare the composition and abundance of functional genes in root-associated microbial communities, no significant differences were found between different varieties or stages (Supplementary Figure 3). The following six COG function classifications were discovered to be directly related to nitrogen-fixation: COG1433, COG2710, COG4656, COG5420, COG5456, and COG5554, with no significant difference between different varieties or sampling stages (Figure 5). However, in the niche RT, the relative abundance of COG5456, also known as nitrogen fixation protein FixH, was higher than in the niches RS, SS, and BS (Figure 5). Lastly, the network complexity of bacterial and fungal communities was unaffected by the host niches of maize rhizosphere (Supplementary Figure 4).

## Discussion

In the present study, the two varieties, control maize line B73 and transgenic line 2A-7, were used as plant material to assess the potential effects of Bt insect-resistant gene on the basic physicochemical properties of plant and rhizosphere soil, the rhizosphere nutrient dynamics, and the composition and abundance of root-associated microbial communities. Our results showed that GM maize only altered the rhizosphere nutrient dynamics, rather than the assembly and shift of microbial communities. This result was consistent with the previous studies, where Bt crop growth had a significant impact on soil enzyme activities (Li et al., 2019; Mandal et al., 2020). In the present study, the enzyme activities of sucrase were significantly higher in 2A-7 groups at the jointing stage compared to control B73 (Figure 1), suggesting that the GM maize increased the fructose and glucose supply in soil, which further led to an increase in carbon cycling. Soil enzymes are a sensitive indicator of soil metabolic processes and fertility condition, and their activities are influenced by soil chemical properties and microbial compositions in GM plants (Bennicelli et al., 2009; Li et al., 2019; Bila et al., 2020). When the high-throughput sequencing results were analyzed, there existed no significant difference between B73 and 2A-7 in the composition and function of root-associated microbial communities. Thus, the Bt maize 2A-7 only altered the rhizosphere nutrient dynamics and had no effect on the microbial community assembly and shift.

Our findings revealed that the differentiation of root-associated microbial communities is primarily determined by host niches rather than the Bt insect-resistant gene or Bt protein secretion. In this study, the nitrogen-fixing bacterial genus *Pseudomonas* (Wen et al., 2019) was found to be enriched in RT, as were three nitrogen-fixing species, *H. huttiense*, *R. leguminosarum*, and *S. azotifigens* (Lakzian et al., 2002; Xie and Yokota, 2006; Andreozzi et al., 2019; Zilli et al., 2020), when compared to RS and SS (Figure 3). The functional prediction also confirmed that FixH nitrogen-fixing protein described as COG5456 was enriched in the niche RT (Figure 5). These results suggest that the niche RT tend to enrich other PGPR, particularly those related to nitrogen-fixation, thereby assisting maize in absorbing nitrogen for growth advantages.

Fungal communities have a wide range of effects on their hosts, ranging from stimulating plant growth to causing diseases (Horbach et al., 2011; Kubicek et al., 2014; Ditengou et al., 2015). One identified beneficial fungus *M. hyalina* was found to be more prevalent in the SS and RS niches than in the RT (Supplementary Table 4). It has been demonstrated that species *M. hyalina* was considered as food to decomposer animals in the soil, and can colonize the roots of *Arabidopsis* and suppress diseases (Maraun et al., 1998, 2003; Johnson et al., 2019). Furthermore, 16 pathogenic fungal species that are detrimental to plant growth were identified in this study (Rojo et al., 2007; Kazan et al., 2012; Carraro et al., 2021), with the relative abundance of maize pathogenic fungus species *G. radicicola* being higher in RS and RT than in SS (Supplementary Table 4). Some symbiotic plant growth promoting fungi (PGPF) may form ideal dispersal networks in the rhizosphere soil to aid in rhizobia enrichment, which may explain the higher relative abundance of *M. hyalina* in the niches SS and RS, while the interaction between non-symbiotic PGPF and plant roots is still poorly understood (Pieterse et al., 2014; Zhang et al., 2020). The recruitment of plant growth-promoting microorganisms and inter-kingdom interactions between bacteria and fungi have a strong influence on the network complexity of microbial communities (Shi et al., 2021), and different endophytic fungi have different colonization abilities in and on the root surface (e.g., endosphere and rhizoplane) (Macia-Vicente et al., 2008). Thus, the assembly of GM maize rhizosphere microbial communities was primarily driven by host niches in the maize rhizosphere, and their mechanism in the distribution of maize rhizosphere microbial communities remains to be investigated further.

The sampling stages, rather than the Bt gene, had a significant effect on the C and N contents of maize, as evidenced by the fact that maturing-stage maize had a higher C content and a lower N content than jointing- and flowering-stage maize (Table 1). When comparing the composition and abundance of maize rhizosphere microbial communities between different sampling stages, the genus *Bacillus* was found to be enriched and the genus *Stenotrophomonas* was found to be depleted at the maturing stage (Figure 3). Some *Bacillus* species belonging to zinc and phosphate solubilization bacteria, which can improve nutrient uptake and maize growth and yield (Mumtaz et al., 2017; Azeem et al., 2022). Additionally, some *Stenotrophomonas* species belonged to the PGPR and were closely associated with plant N, P, and K uptakes (Zhang et al., 2018; Eke et al., 2019). Thus, the differential distribution of these two bacteria at various sampling stages could partially account for the changes in the C and N content of maize. In addition, we were surprised to discover that the prevalence of the maize pathogen that causes stalk rot, *G. radicicola* (Carraro et al., 2021), gradually increased throughout the maize growth cycle (from jointing to flowering to maturation) (Supplementary Table 4). According to a recent study, plant developmental stage primarily drives the differentiation in ecological role of the maize microbiome, for example, the beneficial bacterial taxa such as *Burkholderiaceae* and *Rhizobiaceae* in plant microbiomes were enriched at the early stage, whereas saprophytic fungi were enriched at the later stage (Xiong et al., 2021), which is consistent with our findings.

There are some limitations to the study as well. For example, neither the yield traits of maize nor the residues of Bt protein in soil secreted by Bt maize were detected. Furthermore, plant developmental stages have a greater impact on microbial diversity, composition and interkingdom networks in plant compartments than in soils, especially in the phylloplane (Xiong et al., 2021). Due to the complex network of root-soil-microbe interactions, it is necessary to analyze the effects of growth stages on rhizosphere interactions and functional groups of microbes by including more niches of host plant in further long-term studies in order to obtain comprehensive data for evaluating the bio-safety of the Bt insect-resistant gene on the soil environment in ecological agriculture. In conclusion, there was no discernible impact of transgenic insect-resistant maize 2A-7 on root-associated microbial communities in this short-term study, whereas the composition and function of root microbiota were mainly primarily influenced by rhizosphere niches along the soil-plant continuum.

## Conclusion

Experimental results showed that the transgenic insect-resistant maize line 2A-7 (produced by the insertion of the *mcry1Ab* and *mcry2Ab* genes) only altered the rhizosphere nutrient dynamics, rather than the assembly and shift of microbial communities. Moreover, the host niches and growing stages of maize had greater influence on the assembly and shift of microbial communities than the Bt insect-resistant gene or Bt protein secretion in this short-term study. Thus, the study showed that GM insect-resistant maize can be considered to be safe for the ecosystem functions of the soils, especially for the composition and function of root-associated microbial communities, which will be beneficial for safety control in the application of GM crops in the ecological agriculture.

## Data availability statement

The datasets presented in this study can be found in online repositories. The names of the repository/repositories and accession number(s) can be found below: https://www.ncbi.nlm.nih.gov/ (PRJNA785383 and PRJNA785713).

## Author contributions

YY, XS, and RY designed the experiment. ZW, WY, MH, and XX carried out the experiment. ZW, WY, MH, FW, MY, RY, AF, TY, JQ, and GL analyzed the data. ZW and XX wrote the manuscript. All authors contributed to writing and revision of the manuscript.

## Footnotes

1
http://vdb3.soil.csdb.cn/

2
http://www.arb-silva.de

3
http://unite.ut.ee/index.php

4
https://cloud.majorbio.com/
