# Supplementary material for: Differential assembly of root-associated bacterial and fungal communities of a dual transgenic insect-resistant maize line at different host niches and different growth stages
Source: Front Microbiol. 2022 Sep 29;13:1023971. doi: 10.3389/fmicb.2022.1023971 (PMC9557180; doi:10.3389/fmicb.2022.1023971)
Supplement: Supplementary file 1 [file Data_Sheet_1.docx]

Supplementary Material

**Differentia****l assembly of root-associated bacterial and fungal communities of a dual transgenic insect-resistant maize line at different** **host niches and different growth stages**

Zhongling Wen^1,2^, Weixuan Yao^1^, Mi Han^1^, Xinhong Xu^1^, Fengci Wu^3^, Minkai Yang^1,2^, Aliya Fazal^1^, Tongming Yin^2^, Jinliang Qi^1,2^, Guihua Lu^1,2,4^, Rongwu Yang^1^*, Xinyuan Song^3^*, Yonghua Yang^1,2^*

^1^ Institute for Plant Molecular Biology, State Key Laboratory of Pharmaceutical Biotechnology, School of Life Sciences, Nanjing University, Nanjing 210023, China

^2^ Co-Innovation Center for Sustainable Forestry in Southern China, Nanjing Forestry University, Nanjing 210037, China

^3^ Jilin Provincial Key Laboratory of Agricultural Biotechnology, Agro-Biotechnology Research Institute, Jilin Academy of Agricultural Sciences, Changchun 130033, China

^4^ School of Life Sciences, Huaiyin Normal University, Huaian 223300, China

*** Correspondence:**

Yong-Hua Yang: yangyh@nju.edu.cn, Tel (Fax): 86-25-89686305;
Xinyuan Song: songxinyuan1980@163.com

Rongwu Yang: robertyang@nju.edu.cn

**Key words:** Differential assembly; microbial communities; genetically modified maize; host niches; growing stages

**Running title:** Host niches drove the assembly of GM maize rhizosphere microbial communities

# Supplementary Figures


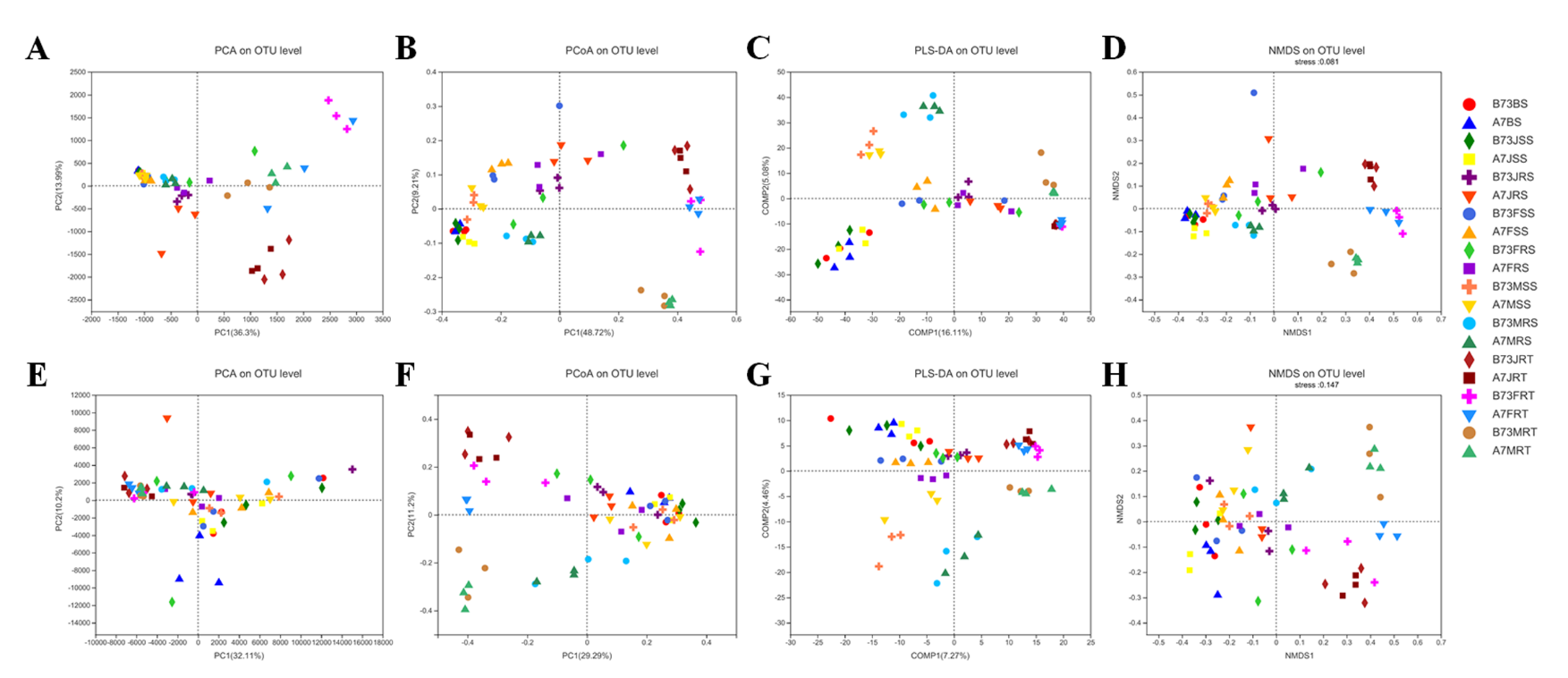


**Fig. S1 PCA, PCoA, PLS-DA and NMDS charts of bacterial community (A, B, C and D) and fungal community (E, F, G and H).** B73 and A7 represent the control maize line B73 and the transgenic insect tolerant maize line 2A-7, respectively. J, F, and M represent the jointing, flowering, and maturing stages of maize, respectively. BS, SS, RS, and RT represent the bulk soil before planting, surrounding soil, rhizospheric soil and root samples, respectively.


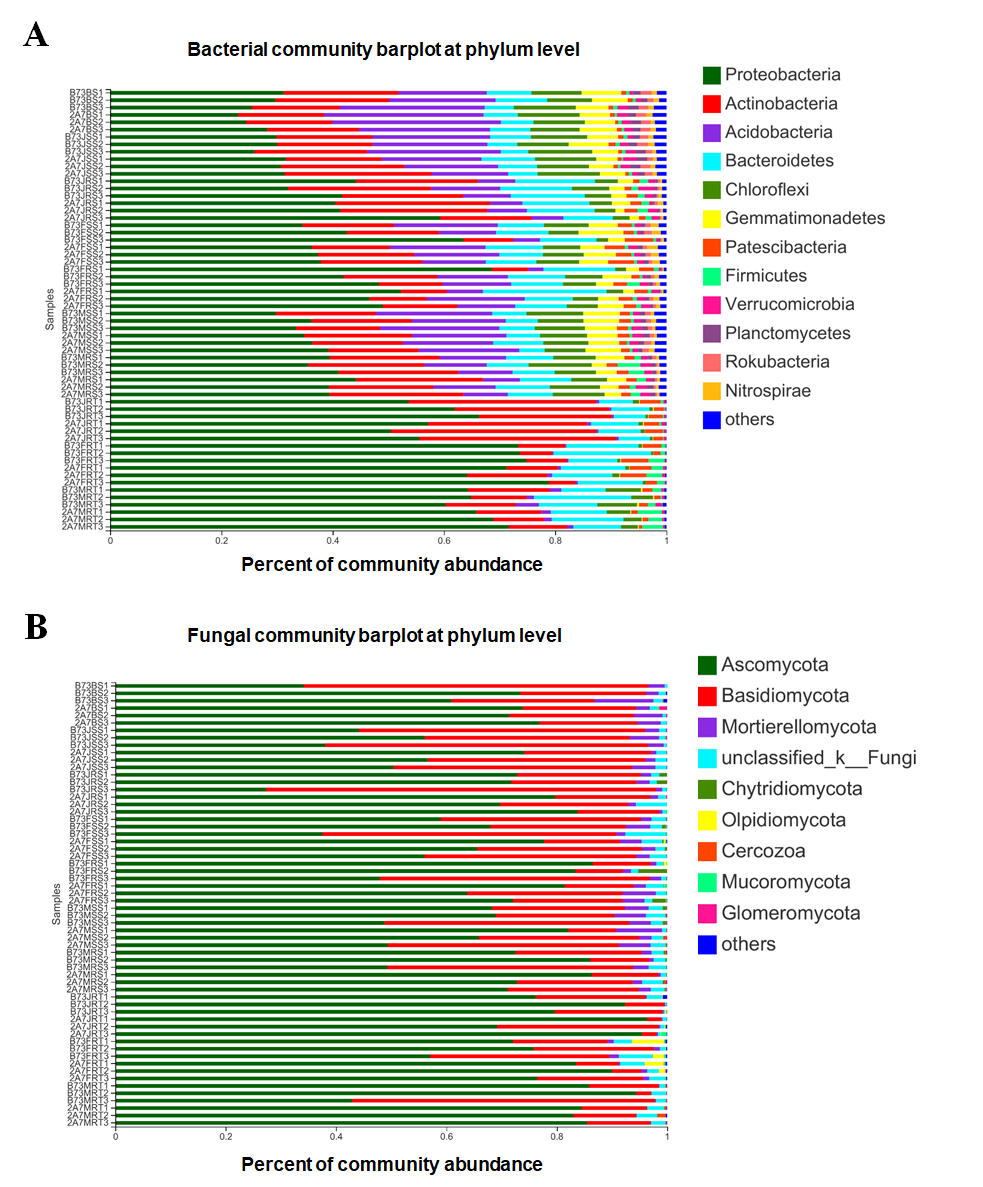


**Fig. S2 Community composition and abundance of each sample at phylum level.** Treatment’s details were as in Fig. 1.


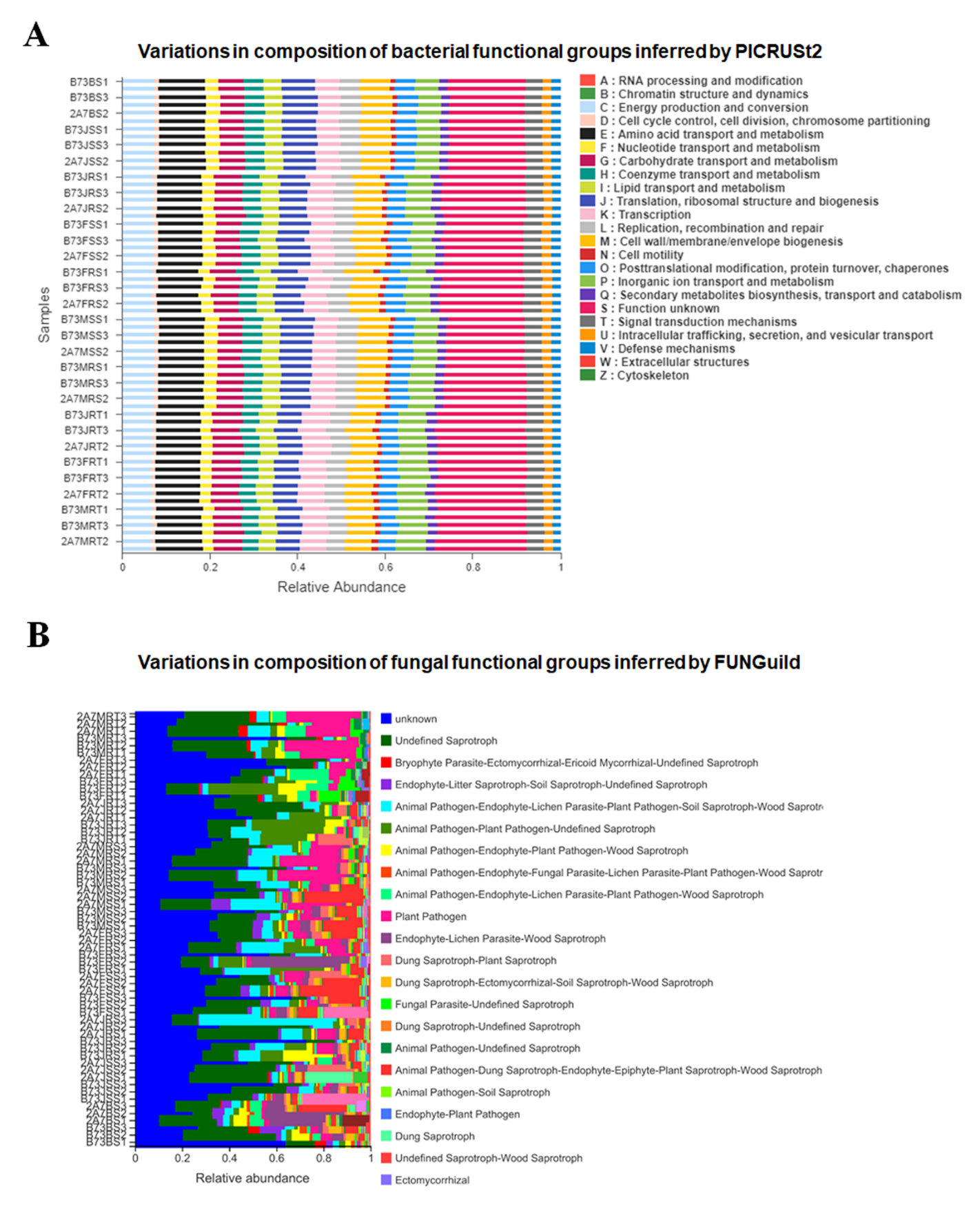


**Fig. S3 Variations in composition of microbial functional groups inferred by PICRUSt2 (A) and FUNGuild (B).** Treatment’s details were as in Fig. 1.


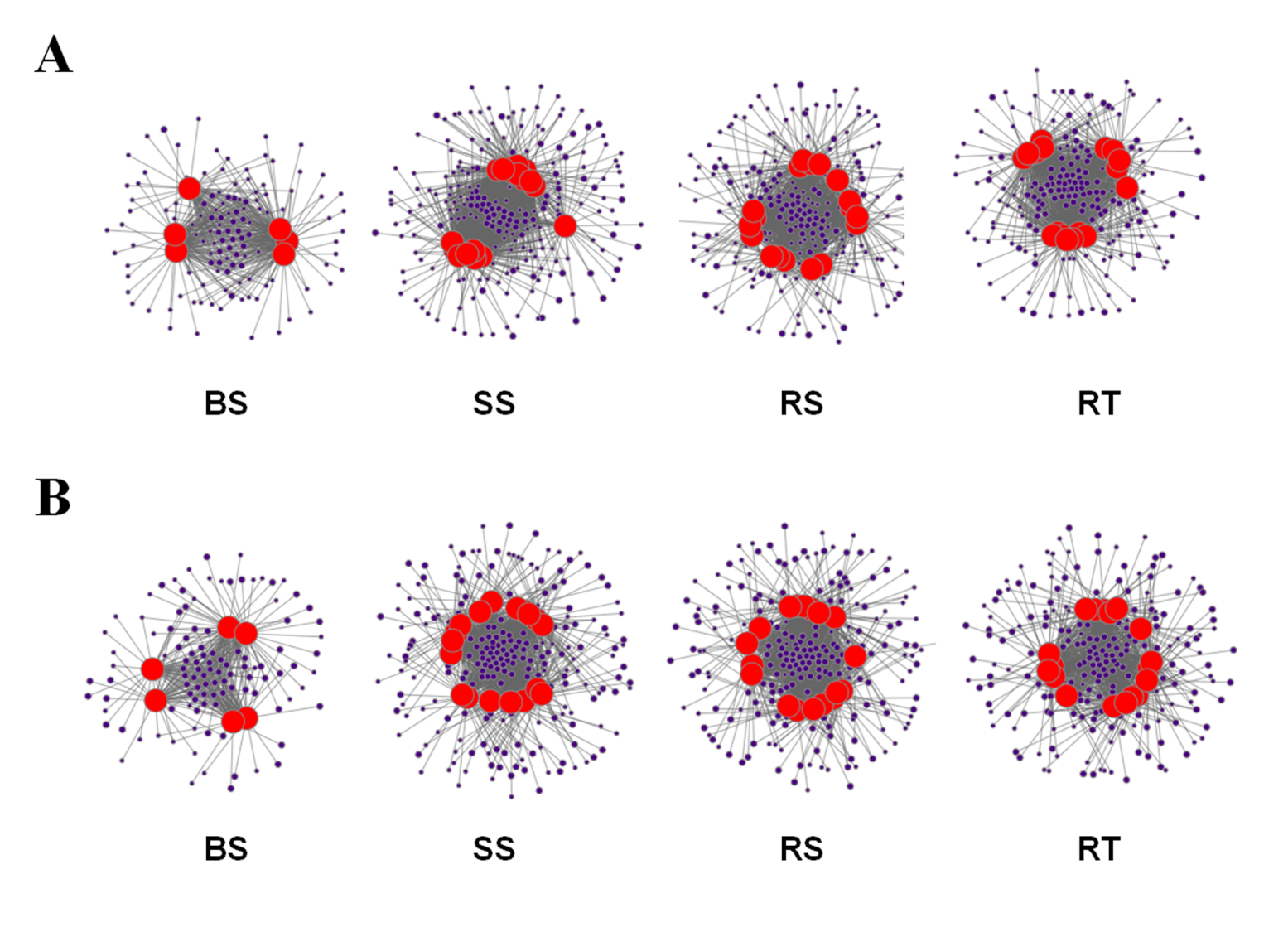


**Fig. S4 Network complexity of maize root-associated bacterial (A) and fungal (B) communities.** BS, SS, RS, and RT represent the bulk soil before planting, surrounding soil, rhizospheric soil and root samples, respectively.
